# Single-Cell Transcriptomic Profiling of Ectopic ACTH-Secreting Pheochromocytoma Reveals the Chromaffin Cell Origin of Ectopic Hormone Production

**DOI:** 10.3390/ijms27083625

**Published:** 2026-04-18

**Authors:** Xu Wang, Penghu Lian, Guoyang Zheng, Wenda Wang, Yang Zhao, Yushi Zhang

**Affiliations:** Department of Urology, Peking Union Medical College Hospital, Chinese Academy of Medical Sciences & Peking Union Medical College, Beijing 100730, China; wangxumedic@163.com (X.W.); lianpenghu@pumch.cn (P.L.); guoyang198966@163.com (G.Z.); wangwenda1989@126.com (W.W.)

**Keywords:** ectopic ACTH syndrome, pheochromocytoma, clinical characteristics, single-cell RNA sequencing, tumor microenvironment, metabolic reprogramming, immunosuppression

## Abstract

Ectopic ACTH-secreting pheochromocytomas are rare and life-threatening endocrine tumors responsible for hypertension, paroxysmal symptoms, and Cushing’s syndrome. The cellular origin of ACTH and the tumor’s molecular characteristics remain poorly understood. Single-cell RNA sequencing was performed on tumor specimens and adjacent adrenal tissues from three patients with ectopic ACTH-secreting pheochromocytomas. Integrated bioinformatic analyses, including differential expression, functional enrichment, cell–cell communication, and pseudotemporal trajectory inference, were conducted. Key findings were supported by immunofluorescence and immunohistochemical staining. Our study integrated single-cell transcriptomic profiling with detailed clinical characterization of three cases of ectopic ACTH-secreting pheochromocytomas. All patients presented classic Cushing’s features and variable catecholamine secretory patterns. Hormone levels improved after surgical resection. Single-cell analysis revealed a complex tumor microenvironment comprising 11 distinct cell populations. Chromaffin cells expressing the ACTH precursor gene POMC were identified within the tumor cell population, suggesting that these cells may represent the source of ectopic ACTH production. This finding was further supported by immunofluorescence and immunohistochemical staining demonstrating ACTH expression in CHGA-positive chromaffin tumor cells and absence of staining for the adrenocortical marker α-inhibin. These tumor cells exhibited metabolic reprogramming characterized by upregulation of oxidative phosphorylation pathways and downregulation of adaptive immune responses. Cell–cell communication analysis suggested interactions between POMC-expressing chromaffin cells and cytotoxic immune cells. Pseudotemporal trajectory analysis further suggested that these chromaffin cells did not transition toward a steroidogenic fate. This study provided a single-cell atlas of ectopic ACTH-secreting pheochromocytomas. Our integrated analysis suggested POMC-expressing chromaffin cells may represent the cellular source of ectopic ACTH production and revealed a transcriptional signature involving metabolic activation and immune modulation that might contribute to tumor progression. These findings offered new insights into the pathophysiology of this rare disease and provided a framework for future investigations into the molecular mechanisms underlying ectopic ACTH production.

## 1. Introduction

Pheochromocytomas (PCC) are rare neuroendocrine tumors arising from chromaffin cells of the adrenal medulla, characterized by their capacity to synthesize and secrete catecholamines, including epinephrine, norepinephrine, and dopamine. Epidemiologically, PCC has an estimated annual incidence of approximately 0.5 cases per 100,000 person-years [1]. The clinical presentation of PCC is highly variable, ranging from asymptomatic incidentalomas to the classic triad of headache, palpitations, and sweating [2,3]. Sustained or paroxysmal hypertension caused by catecholamine excess is a well-established feature of PCCs and can lead to severe cardiovascular complications if not promptly diagnosed and managed [4].

While catecholamine overproduction remains the symbolic endocrine abnormality in PCCs, several subsets exhibited atypical hormonal secretion profiles [5,6,7]. Among these rare variants, ectopic adrenocorticotropic hormone (ACTH)-secreting PCCs represent a special clinical subtype [8,9]. Ectopic ACTH syndrome (EAS), most commonly associated with bronchial, pancreatic neuroendocrine tumors and small cell lung cancer, is an uncommon but life-threatening form of Cushing’s syndrome resulting from non-pituitary ACTH or its precursor pro-opiomelanocortin (POMC) overproduction [10]. When associated with PCCs, EAS manifests as a severe hypercortisolic state characterized by rapid onset of metabolic disturbances such as hypokalemia, hyperglycemia, marked hypertension, truncal obesity, and other symptoms, combining with the clinical presentation of hypercatecholaminemia [11,12].

Ectopic ACTH-secreting pheochromocytomas (eas-PCC) present a significant clinical challenge due to their potential for life-threatening cardiovascular complications and profound metabolic disturbances [13]. There is an urgent need to determine the cellular origin of the hormone and identify the molecular drivers. Although conventional methods could confirm the presence of ACTH within tumor tissues and provide an averaged gene expression profile, the pronounced heterogeneity of pheochromocytoma tissue [14] obscured the critical distinction of whether ACTH is synthesized by the neoplastic chromaffin cells themselves or by other cell clusters within the tumor microenvironment (TME). The lack of cellular resolution fundamentally impedes a deeper understanding of the pathophysiological mechanisms of eas-PCC. Consequently, high-resolution approaches capable of comprehensively characterizing the cellular composition and gene expression networks were chosen to identify the source of ACTH and decipher its regulatory pathway.

In this study, single-cell RNA sequencing (scRNA-seq) was performed on tumor specimens and adjacent adrenal tissues from three patients with eas-PCC. ScRNA-seq enabled a systematic dissection of the TME and transcriptional profiles at the single-cell level [15,16]. Our study aimed to identify the cellular origin of ectopic ACTH production and determine potential regulatory pathways, which provided novel insights into this rare and clinically challenging disease. Given the extreme rarity of eas-PCC, large-scale cohort studies are currently unfeasible. Therefore, this study, although limited to three cases, aims to provide an exploratory single-cell transcriptomic atlas of this entity, and the conclusions of our work still need validation in further studies.

## 2. Results

### 2.1. Baseline and Clinical Characteristics of Eas-PCC Cases

This study included three patients diagnosed with eas-PCC, each presenting with distinct clinical features and secretory patterns (Table 1). Patient A was a 58-year-old female who presented with 1 month of fatigue and lethargy. She had a history of diabetes mellitus. During her clinical course, she suffered Takotsubo cardiomyopathy, which was mistaken for acute coronary stenosis and severe pulmonary infection, caused by elevated ACTH, cortisol, and catecholamine levels. Physical examination revealed striae on the inner thighs. CT showed a large 8 cm left adrenal mass, and ^131^I-MIBG scintigraphy demonstrated intense radiotracer uptake. She has been followed for 5 years postoperatively with sustained normalization of hormone levels and no evidence of recurrence.

Patient B was a 22-year-old female who presented with a 3-month history of weight gain and edema, accompanied by transient loss of consciousness. She had a medical history of diabetes mellitus and osteoporosis. Physical examination revealed moon facies, facial acne, and widespread ecchymoses and striae. Endocrine evaluation demonstrated loss of diurnal cortisol rhythm and non-suppressible ACTH and cortisol levels. However, catecholamine levels were not significantly elevated. CT imaging identified a 2 cm right adrenal mass with enhancement, and ^131^I-meta-iodobenzylguanidine (^131^I-MIBG) scintigraphy showed increased radiotracer uptake in the lesion. She has maintained normal hormone levels for 6 months after surgery.

Patient C, a 42-year-old male, was admitted with a 2-week history of episodic dizziness and headache. He had a history of diabetes mellitus. Physical examination showed scattered ecchymoses on the limbs without other remarkable signs. Endocrine testing confirmed loss of cortisol rhythm, elevated ACTH, and non-suppressible hypercortisolemia. Catecholamine levels were markedly elevated. CT revealed a 4.3 cm right adrenal mass with significant enhancement. ^131^I-MIBG scintigraphy was negative, while somatostatin receptor imaging showed mild uptake in the adrenal lesion. At 5 months of follow-up, he remains biochemically cured.

**Figure 1:**
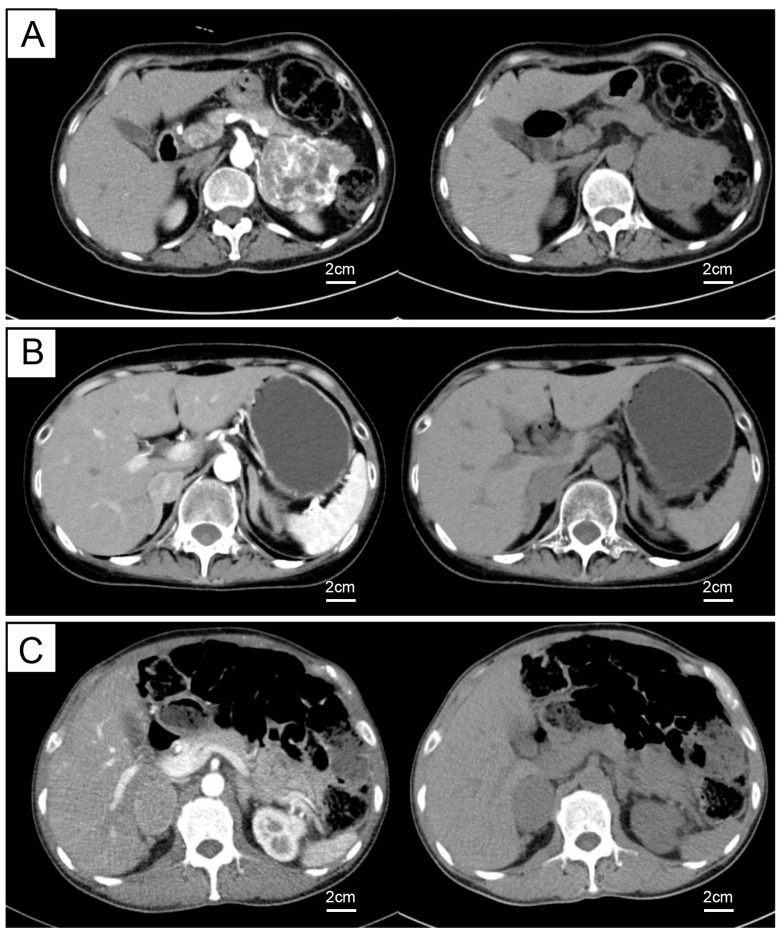
Enhanced CT characteristics of eas-PCC in three cases. (**A**–**C**) Enhanced CT images of eas-PCC in three cases, shown in the arterial phase and unenhanced phase. Scale bar = 2 cm.

The representative CT images of these cases are shown in Figure 1. Following the exclusion of alternative diagnoses, all three patients were diagnosed with eas-PCC and underwent successful surgical resection. The diagnosis was histologically confirmed in all cases. Postoperatively, abnormal hormone levels improved greatly within a week (Table 1). All three patients are still under active follow-up with normal hormone ranges.

### 2.2. Cellular Composition of Ectopic ACTH-Secreting Pheochromocytomas

**Figure 2:**
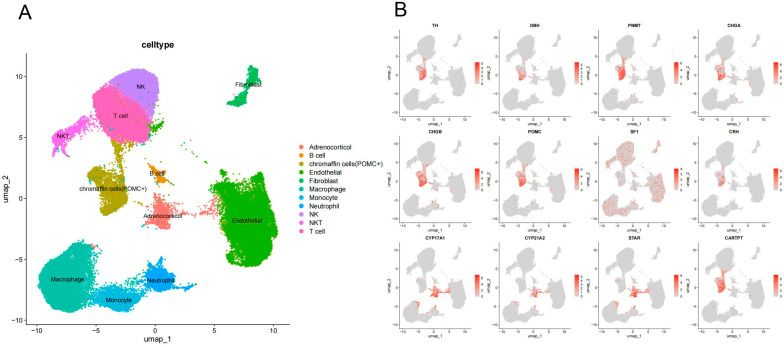
Cellular heterogeneity and key marker expression in eas-PCC. (**A**) Uniform manifold approximation and projection (UMAP) visualization of all cells from three ACTH-secreting pheochromocytomas and matched adjacent adrenal tissues, revealing 11 distinct cell populations. Major cell types were annotated based on canonical marker genes, including adrenocortical cells, chromaffin cells (POMC+), endothelial cells, fibroblasts, macrophages, monocytes, neutrophils, B cells, T cells, NK cells, and NKT cells. (**B**) Feature plots showing the expression of representative marker genes across the UMAP. Chromaffin cell lineage markers (TH, DBH, PNMT, CHGA, and CHGB) were enriched in the tumor-associated neuroendocrine cell population. Expression of POMC, the precursor gene of ACTH, was detected within the CHGA-positive cell cluster. Adrenocortical cells were characterized by steroidogenic markers including SF1, CYP17A1, CYP21A2, and STAR. Immune and stromal cell populations were identified based on lineage-specific transcriptional signatures. Abbreviations: SF1: steroidogenic factor 1; CRH: corticotropin-releasing hormone; STAR: steroidogenic acute regulatory protein; CYP21A2: cytochrome P450 family 11 subfamily A member 2; CYP17A1: cytochrome P450 family 17 subfamily A member 1; CARTPT: cocaine- and amphetamine-regulated transcript propeptide.

Single-cell RNA sequencing was performed on tumor specimens and adjacent adrenal tissues from three patients with eas-PCC. Clustering of the combined dataset revealed 11 distinct cell populations, as visualized by UMAP projection (Figure 2A). The distribution of tumor and adjacent adrenal cells within the integrated UMAP space is shown in Supplementary Figure S2. The tumor-derived chromaffin cells exhibited a high expression of neuroendocrine markers such as Chromogranin A (CHGA), Chromogranin B (CHGB), Tyrosine Hydroxylase (TH), Dopamine Beta-Hydroxylase (DBH), and Phenylethanolamine N-Methyltransferase (PNMT), as well as POMC (the precursor of ACTH), which was named “chromaffin cells (POMC+) or POMC-expressing chromaffin cells” (Figure 2B), suggesting that these tumor-derived chromaffin cells may represent the source of ectopic ACTH production. And further studies will be required to clarify the mechanisms of POMC processing in these cells. The remaining clusters were determined based on established marker genes and were annotated as follows: adrenocortical cells, B cells, endothelial cells, fibroblasts, macrophages, monocytes, neutrophils, natural killer (NK) cells, NKT cells, and T cells. Detailed feature plots are presented in Supplementary Figure S3. The comprehensive cellular composition highlighted the heterogeneous nature of the tumor microenvironment in eas-PCC.

### 2.3. Functional Enrichment of Ectopic ACTH-Secreting Pheochromocytomas

**Figure 3:**
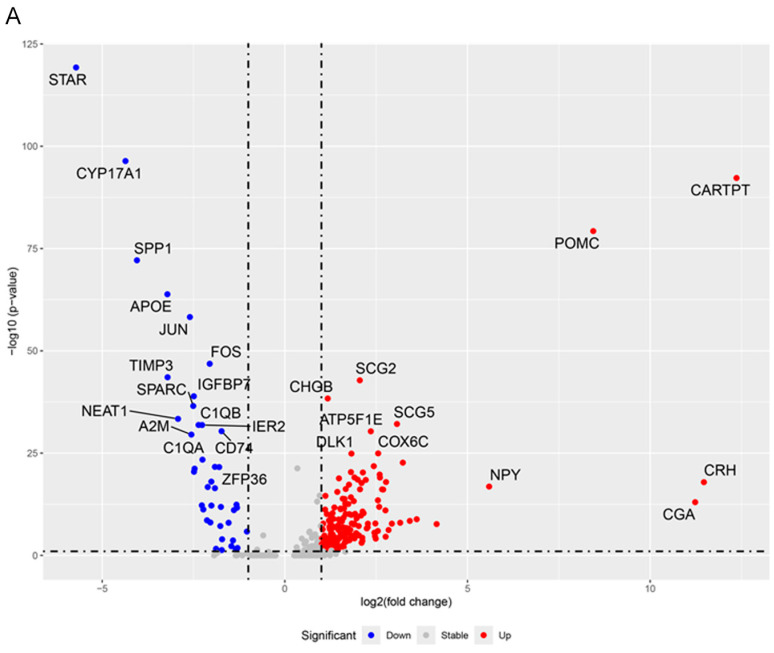

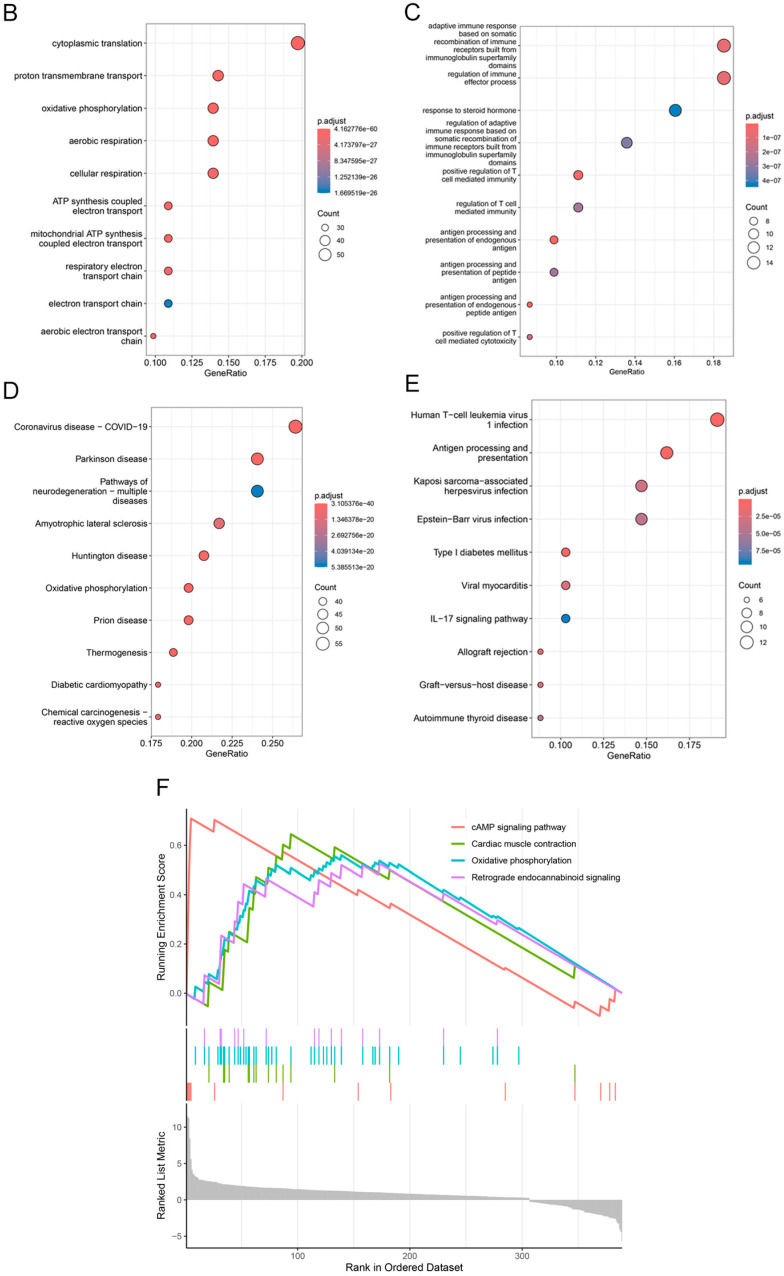
Differential gene expression and functional enrichment of eas-PCC. (**A**) Volcano plot displaying differentially expressed genes between tumor and normal CHGA^+^ chromaffin cells. A total of 170 genes were significantly upregulated (red), and 41 genes were significantly downregulated (blue) (adjusted *p*-value < 0.05, |log2 fold change| > 1). Key neuroendocrine markers were labeled. (**B**,**C**) GO biological process enrichment analysis for upregulated (**B**) and downregulated (**C**) genes. The top significantly enriched terms are shown. (**D**,**E**) KEGG analysis for upregulated (**D**) and downregulated (**E**) pathways. (**F**) GSEA plot showing the top four enriched gene sets.

To clarify the specific pathogenic mechanisms of ectopic ACTH secretion, chromaffin cells were isolated for further differential expression analysis. Comparison between tumor and normal groups revealed a distinct transcriptional profile, with 170 genes significantly upregulated and 41 genes significantly downregulated in tumor chromaffin cells (POMC+) (adjusted *p*-value < 0.05, |log2 fold change| > 1), as visualized in the volcano plot (Figure 3A). As expected, classic neuroendocrine markers including CHGB, POMC, Secretogranin II (SCG2), and Secretogranin V (SCG5) were significantly upregulated in the tumor cells, suggesting that these tumor cells may represent the source of ectopic ACTH.

GO biological process analysis of the upregulated genes in tumor chromaffin cells (POMC+) revealed a pronounced enrichment in processes related to energy metabolism, including “oxidative phosphorylation,” “aerobic respiration,” “cellular respiration,” and “ATP synthesis” (Figure 3B). Conversely, downregulated genes were significantly associated with immunosuppression, such as “adaptive immune response,” “regulation of T cell mediated cytotoxicity,” and “antigen processing and presentation” (Figure 3C). This enrichment pattern was consistent with KEGG pathway analysis (Figure 3D,E). To further validate these findings without pre-defined thresholds, Gene Set Enrichment Analysis (GSEA) was performed. The pathway of “Oxidative phosphorylation” was ranked among the top four most significantly enriched gene sets (Figure 3F). Collectively, these results indicated that eas-PCC underwent enhanced energy production while evading immune surveillance.

### 2.4. Cell–Cell Communication Network in the TME

**Figure 4:**
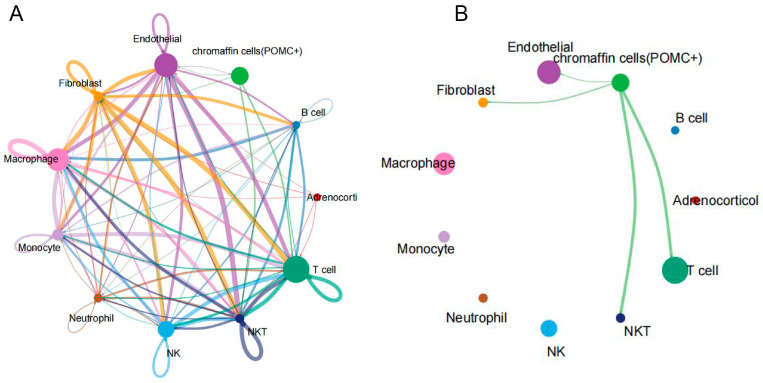
Cell–cell communication analysis between cell clusters in eas-PCC. (**A**) Circos plot depicting the overall intercellular communication network among all 11 clusters. The width of the edges represents the interaction strength between cell types. (**B**) Specific communication patterns between chromaffin cells (POMC+) and four selected cell types (CD8^+^ T cells, NKT cells, endothelial cells, and fibroblasts). The thickness of the lines corresponded to the number of significant ligand-receptor interactions.

To systematically present the intercellular signaling network within the tumor microenvironment of eas-PCC, CellChat computational analysis was applied to the 11 identified cell clusters. The comprehensive cell–cell communication network across all clusters revealed extensive and complex interaction patterns among chromaffin cells (POMC+), stromal, and immune cells (Figure 4A). The cell–cell communication of each cluster was shown in Supplementary Figure S4.

Notably, when focusing specifically on outgoing and incoming signals of chromaffin cells (POMC+), distinct communication patterns with key components were observed. As shown in Figure 4B, the tumor cells exhibited particularly strong interaction strengths with CD8^+^ T cells and NKT cells, as evidenced by substantially thicker lines than those with endothelial cells and fibroblasts. This pattern suggested a potent and specific connection between tumor cells and cytotoxic lymphocyte populations.

### 2.5. Distinct Hormonal Signature and Pseudotemporal Trajectory of POMC-Expressing Chromaffin Cells

**Figure 5:**
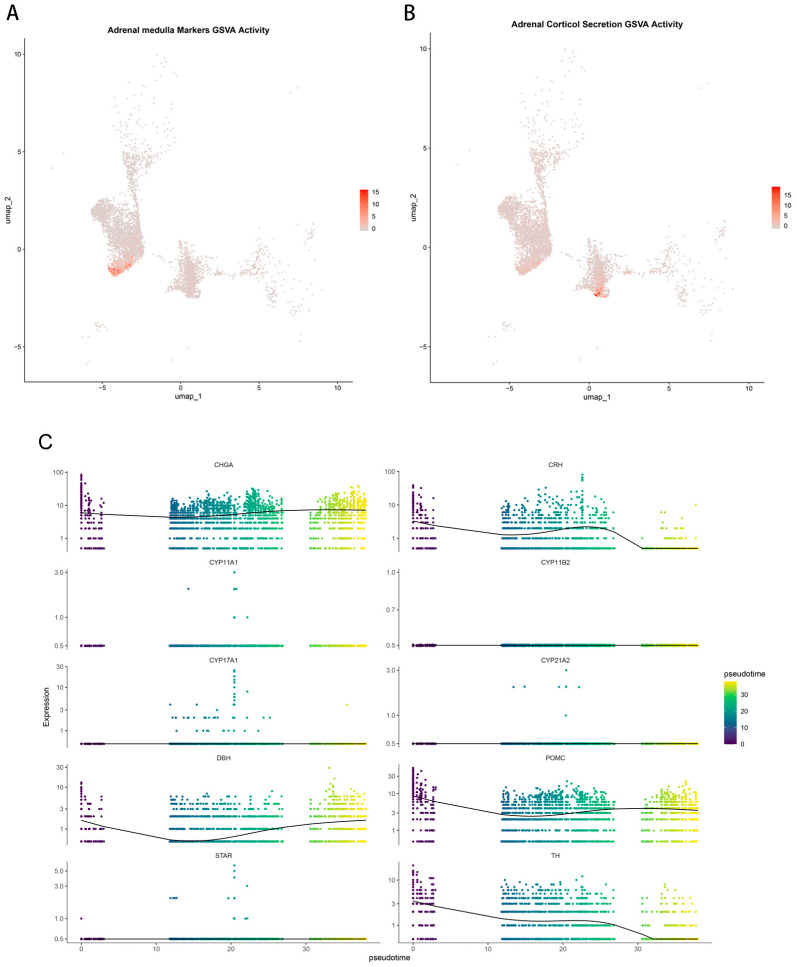
Functional identity and lineage stability of tumor cells. (**A**) GSVA enrichment scores for catecholamine biosynthesis gene sets in chromaffin cells (POMC+). (**B**) GSVA enrichment scores for corticosteroid biosynthesis gene sets in the adrenocortical cells. (**C**) Pseudotemporal trajectory analysis of chromaffin cells (POMC+). Pseudotime trajectory analysis of these chromaffin cells shows dynamic expression of key neuroendocrine markers (e.g., CHGA, DBH, TH, and POMC) and steroidogenic genes (e.g., CYP11A1, CYP11B2, and STAR).

To evaluate and compare the hormonal synthesis capacities, Gene Set Variation Analysis (GSVA) was applied to the chromaffin cells (POMC+) and adrenocortical cells. The catecholamine signature encompassed key synthetic enzymes and neurosecretory markers, while the corticosteroid signature included critical steroidogenic enzymes and regulators (Supplementary Table S1). GSVA revealed a clear functional difference: chromaffin cells (POMC+) exhibited high enrichment scores for the catecholamine synthesis pathway but showed no activity in the corticosteroid signature (Figure 5A). Conversely, adrenocortical cells displayed strong enrichment for corticosteroid biosynthesis without any significant activity in catecholamine production (Figure 5B). These results indicated that catecholamines in these tumors were derived from the chromaffin cell lineage, while steroid hormone production was confined to the adrenocortical cells.

To further explore transcriptional dynamics associated with ectopic ACTH production, pseudotemporal trajectory analysis was performed on chromaffin cells using a panel of key lineage markers. The inferred trajectory revealed that canonical chromaffin markers, together with POMC, were broadly expressed across the pseudotime axis. In contrast, key steroidogenic enzymes remained absent throughout the trajectory (Figure 5C). These findings suggest that ectopic ACTH production represents a stable transcriptional feature of chromaffin tumor cells rather than reflecting a transition toward a steroidogenic cellular state.

### 2.6. Immunohistochemical and Immunofluorescence Validation Suggested ACTH and CHGA Co-Expression in Tumor Cells

**Figure 6:**
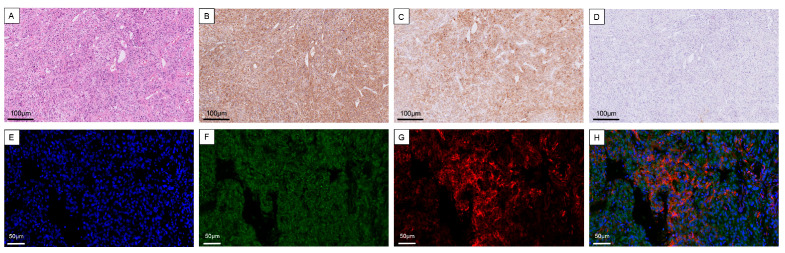
Immunohistochemical and immunofluorescence validation of ACTH expression in chromaffin tumor cells. Representative images from three independent tumor samples are shown. (**A**) Hematoxylin and eosin (H&E) staining showing the typical nested architecture of pheochromocytoma tumor cells. (**B**) Immunohistochemical staining demonstrating strong CHGA expression in tumor cells, confirming their neuroendocrine chromaffin origin. (**C**) Immunohistochemical staining demonstrating ACTH immunoreactivity in tumor cells. (**D**) Immunohistochemical staining showing negative α-inhibin expression in tumor cells, supporting a non-adrenocortical origin. Scale bar = 100 μm. (**E**–**H**) Immunofluorescence staining and confocal microscopy demonstrating co-localization of CHGA (green) and ACTH (red) within the same tumor cells. (**E**) DAPI nuclear staining (blue). (**F**) CHGA staining (green). (**G**) ACTH staining (red). (**H**) Merged image showing partial co-localization of CHGA and ACTH signals. Scale bar = 50 μm.

To provide protein-level evidence supporting the transcriptomic findings, immunohistochemical staining was first performed on surgical eas-PCC specimens. As shown in Figure 6, ACTH immunoreactivity was detected in tumor cells with characteristic chromaffin morphology. CHGA immunostaining further confirmed the neuroendocrine identity of these tumor cells. In contrast, immunostaining for the adrenocortical marker α-inhibin was negative in tumor cells, further supporting a non-adrenocortical origin of ectopic ACTH production.

To further evaluate the spatial relationship between ACTH and chromaffin tumor cells, immunofluorescence staining was performed on tumor sections. Cytoplasmic staining for ACTH (red) was detected in tumor cells that also expressed the neuroendocrine marker CHGA (green), and partial co-localization of these signals was observed in a subset of tumor cells. These findings were consistent with the single-cell RNA sequencing results, showing POMC expression within the chromaffin cell population.

## 3. Discussion

Our study built upon and extended previous single-cell transcriptomic efforts in PCCs. While the seminal work by Zhang et al. offered a broad atlas of cellular heterogeneity of PCCs [17], our analysis provided deep insight into a rare and distinct subtype—the ectopic ACTH-secreting PCC. Ectopic ACTH-secreting PCC represented a rare clinical entity, with current evidence largely derived from case reports. In systematic reviews, Cushing’s syndrome was reported in more than 80% of patients, and infection was the most common complication [7,13]. In our study, Cushing’s signs, diabetes mellitus, and severe pulmonary infection were observed, which underscored the profound metabolic disturbances caused by hypercortisolism. Notably, the clinical presentation of eas-PCC varied greatly. This phenotypic diversity posed great challenges in diagnosis, which meant the necessity of a comprehensive evaluation including both biochemical tests and imaging examinations.

According to the reports, most of the eas-PCC cases who received adrenalectomy achieved biochemical cure from both catecholamine and glucocorticoid excess [7,13]. In our series, all three patients underwent successful surgical resection following appropriate preoperative management of hypercortisolism and hypercatecholaminemia. The improvement of hormone levels and clinical symptoms in all three cases emphasized complete surgical resection as the key point for this complex condition.

The application of scRNA-seq to eas-PCC showed a high-resolution view of their cellular compositions and molecular signatures. Our analysis not only uncovered the heterogeneous TME but also pointed to tumor-derived chromaffin cells as the likely source of ectopic ACTH production. Beyond this central finding, the transcriptomic profile of these cells revealed profound metabolic reprogramming and altered immune-related pathways, suggesting transcriptional programs associated with tumor adaptation and an immunosuppressive microenvironment. Furthermore, pseudotemporal trajectories provided additional insight into the tumor’s current state.

First, scRNA-seq revealed a highly complex TME composed of 11 distinct cell populations, including chromaffin cells (POMC+), adrenocortical cells, fibroblast, and multiple immune cell subtypes. The identification of these populations was robustly supported by established marker genes [18,19]. This heterogeneous diversity underscores the potential for multiple interactions that may influence tumor behavior and hormone secretion. Notably, epithelial cells were absent from all cellular components identified in our study. This finding was consistent with the category mentioned in the 2022 WHO Classification of Paragangliomas and Pheochromocytomas, which confirmed its non-epithelial origin [20].

Our transcriptomic data revealed a co-occurrence of transcriptional programs suggestive of metabolic and immune-altered states within the chromaffin cells (POMC+). Transcriptomic analyses consistently demonstrated a significant upregulation of oxidative phosphorylation (OXPHOS) and related energy metabolism pathways. While this finding appeared to contrast with the classical Warburg effect observed in many proliferative carcinomas [21], it might represent a proper adaptation to the specific physiological demands of neuroendocrine tumor cells [22]. The diverse genetic backgrounds and secretion patterns may match differential metabolism [23,24]. The energy-intensive processes of hormone synthesis and secretion create an exceptionally high demand for ATP, which was likely met by mitochondrial OXPHOS [25]. Concurrently, a marked downregulation of genes towards adaptive immune responses, T cell cytotoxicity, and antigen presentation was observed. This downregulation of immune-related genes was consistent with transcriptional signatures observed in other immunosuppressive tumor environments [26]. Notably, emerging research indicated that tumor-mediated metabolic changes could suppress antitumor immunity by creating a competing TME or through other signaling mechanisms [27]. To support the immunosuppressive phenomenon inferred from transcriptomic data, CellChat was employed—a computational tool that systematically inferred and analyzed cell–cell communication networks by integrating scRNA-seq data with a curated database of ligand-receptor interactions [28]. Our CellChat analysis revealed prominent ligand-receptor mediated interactions between chromaffin cells (POMC+) and CD8^+^ T/NKT cells. While such a connection might be consistent with an immunosuppressive microenvironment, as inferred from the downregulation of adaptive immune genes in tumor cells, it was crucial to note that CellChat only predicted potential communication, and causal relationships remain to be established in further validations.

In order to identify the hormone secretion pattern, GSVA and pseudotemporal trajectory analysis were performed. The results demonstrated that tumor-derived CHGA+ cells stably co-expressed key enzymes (CHGA, DBH, TH) and POMC (the precursor to ACTH), while completely lacking the steroidogenic enzymes that were present only in adrenocortical cells. This expression profile supported the conclusion that these cells were specialized for concurrent catecholamine and ectopic ACTH synthesis. However, the observed functional maturity of the tumor cells prevented further exploration by pseudotemporal trajectory. It remained a pivotal unanswered question whether POMC expression was an early initiating event or a secondary acquisition during tumor progression [29]. It is important to note that pseudotime analysis in this context reflects transcriptional similarity along a continuum of cell states rather than actual chronological differentiation history. Future studies will be required to delineate the development of these molecular events from their preclinical stage.

The immunohistochemical analyses provided protein-level evidence supporting chromaffin tumor cells as the likely cellular source of ectopic ACTH production in eas-PCC. The combined ACTH positivity, CHGA expression, and absence of α-inhibin staining further supported a chromaffin rather than adrenocortical origin of hormone production. Consistent with these findings, immunofluorescence analyses demonstrated co-localization of CHGA and ACTH signals in a subset of tumor cells, providing further support for the involvement of chromaffin tumor cells in ectopic ACTH production. These observations were also in agreement with the single-cell RNA sequencing data, showing POMC expression within the chromaffin cell population. Nevertheless, because the present study combined transcriptomic and protein-level localization analyses rather than direct functional assays of hormone secretion, these findings should be interpreted as supportive rather than definitive evidence of ectopic ACTH production at the single-cell level. Further functional studies will be required to directly demonstrate hormone secretion from individual chromaffin tumor cells.

Our findings may have clinical implications. By showing that ectopic ACTH production likely originated from chromaffin tumor cells rather than from a steroidogenic adrenal lineage, this study provides a cellular framework for understanding hormone dysregulation in pheochromocytoma. From a clinical perspective, these results offer additional cellular-level evidence consistent with the current understanding that surgical tumor removal, when feasible, represents an important strategy for improving hormone excess in affected patients. Furthermore, these findings may inform future studies aimed at identifying molecular pathways that regulate POMC expression and neuroendocrine hormone production, which may contribute to the development of targeted therapeutic approaches for hormone-secreting tumors.

This study has several limitations. First, the cohort size was relatively small (n = 3), which limited the statistical power of differential expression and cell–cell communication analyses. This reflects the inherent difficulty of studying such an ultra-rare disease. Larger cohorts and multi-center collaborations will be necessary to validate the cellular composition and interaction networks identified here. Second, the control samples were obtained from adjacent adrenal tissues rather than completely normal adrenal glands. Because chronic ACTH excess may induce adrenal cortical hyperplasia and transcriptional changes, these tissues may not fully represent normal adrenal physiology. Nevertheless, residual adrenal medullary components were identified macroscopically during specimen processing and were further supported by the detection of chromaffin cell populations expressing canonical lineage markers in the single-cell RNA sequencing data. Finally, although computational analyses were complemented by immunofluorescence and immunohistochemical staining, this study remains primarily descriptive, and further functional studies are required to clarify the mechanisms underlying ectopic ACTH production in pheochromocytoma.

## 4. Materials and Methods

### 4.1. Clinical Specimen Collection

Our study included a total of three patients with pathologically confirmed eas-PCC, along with adjacent adrenal tissues, who underwent surgery at Peking Union Medical College Hospital (PUMCH) between 2021 and 2024. Representative gross specimen images illustrating the tumor and adjacent adrenal tissue are shown in Supplementary Figure S1. All patients exhibited hypersecretion of both ACTH and catecholamines. Written informed consent was obtained from all three patients, and the study protocol was approved by the ethics committee of PUMCH (I-23PJ912). The hormone levels of all patients were back to normal postoperatively. All three fresh tumor tissues and residual adjacent adrenal tissue were carefully identified by gross examination and sampled separately for single-cell dissociation within 30 min after surgical resection. The adjacent adrenal tissue was selected based on its typical anatomical location and gross appearance, including centrally located brown to reddish-brown regions consistent with the adrenal medulla. The histological presence of medulla in adjacent adrenal tissue was identified by experienced pathologists.

### 4.2. Tissue Dissociation and Preparation of Single-Cell Suspensions

The obtained tissues were transferred to a Petri dish (Petri dish was placed on wet ice) pre-spiked with 1 × PBS (without RNase and Ca, Mg ions) and washed with 1 × PBS to remove blood stains, grease, and other adherents from the tissue surface. The tissue was then cut into 0.5 mm^2^ pieces and washed again with 1 × PBS. The washed pieces were added with dissociation solvent (0.35% collagenase IV(Worthington Biochemical, Lakewood, NJ, USA), 2 mg/mL papain(Sigma-Aldrich, St. Louis, MO, USA), 120 Units/mL DNase I (Roche, Basel, Switzerland)) and reacted for 20 min at 37 °C in a water bath shaker (100 rpm). PBS (containing 10% fetal bovine serum) was used to terminate the dissociation. The cells were repeatedly puffed 5–10 times with a pipette gun (gentle handling to prevent cell death due to shear force). The cell suspension was filtered through a 70–30 μm cell sieve and subsequently centrifuged at 300× *g* for 5 min at 4 °C. After centrifugation, the cell sediment was collected, and the cells were resuspended by adding 100 μL of 1× PBS containing 0.04% BSA (Gibco, Grand Island, NY, USA). To remove erythrocytes, 1 mL of 1× erythrocyte lysis solution (MACS Red Blood Cell Lysis Solution, Cat. No. 130-094-183, Miltenyi Biotec, Bergisch Gladbach, Germany) was added and reacted at room temperature or on wet ice for 2–10 min. After lysis, the cells were centrifuged at 300× *g* for 5 min at 4 °C, and the cell pellet was collected after centrifugation. Add 100 μL of Dead Cell Removal MicroBeads (MACS 130-090-101; Miltenyi Biotec, Bergisch Gladbach, Germany), mix well, and incubate for 15 min at room temperature. At the end of incubation, Binding Buffer was added after MS Columns (130-042-201) to remove reagents and dead cells. Cell precipitates were collected by centrifugation at 300× *g* for 5 min at 4 °C. Subsequently, 1 × PBS (0.04% BSA) was used to resuspend the cell precipitate and centrifuged at 4 °C at 300× *g* for 5 min (repeated twice). The candidate cells were obtained after tissue dissociation, lysis of erythrocytes, and removal of dead cells, and 100 μL of 1 × PBS (0.04% BSA) was added to form a cell suspension. The cell activity was detected by the trypan blue staining method, and the cell activity was required to be >85%. The number of cells was counted using a hemocytometer or Countess II Automated Cell Counter, and the cell concentration was 700–1200 cells/μL.

### 4.3. Chromium 10x Genomics Library and Sequencing

A single-cell suspension was added to the 10× Chromium chip according to the instructions for the 10X Genomics Chromium Single-Cell 3′ kit v3(10× Genomics, Pleasanton, CA, USA), with the expectation of capturing 8000 cells. cDNA amplification and library construction were performed according to standard protocols. Libraries were sequenced by LC-Bio Technology on an Illumina NovaSeq 6000 sequencing system (Illumina, San Diego, CA, USA) using paired-end sequencing (150 bp). The sequencing read structure consisted of Read1 (26 bp, containing cell barcode and UMI) and Read2 (98 bp, transcript sequence), with an average depth of approximately 20,000 reads per cell.

### 4.4. Bioinformatics Analysis

Raw sequencing data were processed using Cell Ranger (v7.0.0) for alignment, barcode counting, and unique molecular identifier (UMI) quantification against the GRCh38 reference genome. The resulting gene-cell count matrices for all samples were imported into Seurat (v5.3.0) for integrated analysis. Quality control (QC) was performed per cell using the following thresholds: cells with <500 detected genes or >25% mitochondrial RNA content were considered low-quality and removed. Doublet detection was performed using DoubletFinder (v2.0.3) with an expected doublet formation rate of 8%, and predicted doublets were excluded. To mitigate batch effects across the three patients and between tumor/normal tissues, data integration was performed using the FindIntegrationAnchors and IntegrateData functions in Seurat, selecting 2000 integration anchors. Post-integration, the data were normalized using the LogNormalize method and scaled. Principal component analysis (PCA) was conducted on the scaled data of highly variable genes. Highly variable genes were identified using the FindVariableFeatures function in Seurat. The top 20 principal components, determined by inspection of the elbow plot, were used for neighborhood graph construction and graph-based clustering using the Louvain algorithm at a resolution of 0.8. Cell type annotation was performed by examining the expression of canonical marker genes for each cluster. Differential expression analysis between tumor-derived and normal CHGA+ chromaffin cells was performed using the Wilcoxon rank-sum test implemented in the FindMarkers function, with genes considered significant at an adjusted *p*-value (Bonferroni correction) < 0.05 and absolute log2 fold change > 1.

Cell clusters were annotated by classic markers (Supplementary Table S2). Chromaffin cells and adrenocortical cells were distinguished: chromaffin cells expressed high levels of CHGA, CHGB, TH, and DBH but lacked steroidogenic enzymes such as CYP11B2 and STAR; conversely, adrenocortical cells expressed steroidogenic enzymes but not neuroendocrine chromaffin markers. Clusters expressing ambiguous or mixed marker profiles were not observed, and thus none were excluded.

### 4.5. Gene Ontology and Pathway Enrichment Analysis

Functional enrichment analysis of differentially expressed genes (DEGs) was performed to interpret their biological implications. Gene Ontology (GO) and Kyoto Encyclopedia of Genes and Genomes (KEGG) pathway analysis were conducted using the clusterProfiler package (v4.6.2) in R. The list of Entrez Gene IDs for the significant DEGs (with a threshold of adjusted *p*-value < 0.05 and |log2(Fold Change)| > 1) was used as the input. The organism annotation package (org.Hs.eg.db for Homo sapiens) was employed as the reference background. The enrichGO and enrichKEGG functions were executed. The results were filtered to remove overly broad terms, and significantly enriched terms were defined as those with a corrected *p*-value (FDR) of less than 0.05. The top 10 enriched terms were selected and summarized for presentation in the manuscript.

### 4.6. CellChat Analysis of Cell–Cell Communication

CellChat (v1.6.1) was employed to infer intercellular communication between cell clusters by integrating gene expression data with a curated ligand-receptor interaction database. The normalized expression matrix and cell cluster annotations were used to create a CellChat object. The default CellChatDB.human database was subset to focus on secreted signaling pathways. Overexpressed ligands and receptors were identified using “identifyOverExpressedGenes”, followed by communication probability calculation via “computeCommunProb” with default parameters. Probabilities were filtered, requiring at least 10 cells per cluster. Results were aggregated at the pathway level using “computeCommunProbPathway” and integrated across clusters with “aggregateNet”. All visualizations were generated using built-in CellChat plotting functions.

### 4.7. Pseudotemporal Trajectory Analysis

Pseudotemporal ordering of POMC-expressing chromaffin cells was performed using Monocle 3 (version 1.3.4) to explore transcriptional progression and characterize the dynamic expression patterns of key lineage-related genes. The analysis followed the standard Monocle 3 workflow. After dimensionality reduction and UMAP embedding, a principal trajectory graph was constructed using the learn_graph function. Cells were then ordered along the trajectory using the order_cells function, with the root state determined automatically by Monocle3 based on the structure of the learned trajectory graph. Gene expression dynamics along pseudotime were subsequently visualized to examine changes in neuroendocrine and steroidogenic marker genes during chromaffin cell state transitions.

### 4.8. Immunofluorescence Staining

Immunofluorescence (IF) staining was performed on 4 μm formalin-fixed, paraffin-embedded (FFPE) tumor sections to validate the co-expression of ACTH and CHGA according to standard protocols, which provided supportive evidence for hormone production at the protein level. Sections were incubated overnight at 4 °C with primary antibodies against ACTH (Servicebio, GB14001; Wuhan, China) and CHGA (Servicebio, GB111316; Wuhan, China). After washing, bound antibodies were detected using CY3-conjugated goat anti-mouse IgG and Alexa Fluor 488-conjugated goat anti-rabbit IgG. Nuclei were counterstained with DAPI. Images were acquired using a confocal laser scanning microscope.

The research was developed without the help of artificial intelligence (AI) [30].

### 4.9. Immunohistochemistry Staining

Immunohistochemical (IHC) staining was performed on 4 μm formalin-fixed, paraffin-embedded (FFPE) tumor tissue sections according to standard protocols. After deparaffinization and antigen retrieval, sections were incubated overnight at 4 °C with primary antibodies against ACTH (Servicebio, GB14001), CHGA (Servicebio, GB111316), and α-inhibin (Servicebio, GB11741). Following incubation with HRP-conjugated secondary antibodies, signals were visualized using DAB chromogen and counterstained with hematoxylin. Negative control sections processed without primary antibodies were included to confirm staining specificity. Images were acquired using a bright-field microscope.

### 4.10. Statistical Analysis

All statistical analyses and data visualizations were performed in R (version 4.3.1). Statistical significance was defined as adjusted *p* < 0.05 unless otherwise specified.

## 5. Conclusions

In conclusion, our study provided evidence suggesting that tumor-derived chromaffin cells in eas-PCC were the likely source of ectopic ACTH production. The transcriptomic analysis revealed upregulation of oxidative phosphorylation pathways, which met the high secretory demands. The downregulation of immune processes, as well as inferred communication networks between tumor cells and cytotoxic lymphocytes, provided a reasonable explanation for immunosuppression. Our study substantially advanced the pathophysiological understanding of eas-PCC and provided the potential for developing targeted therapies for this rare tumor entity.

## Figures and Tables

**Table 1:** Hormone results before and after surgery.

Case	Laboratory Results	Value		
		Before Surgery	After Surgery	Reference
A	24-h CAs in urine(μg/24 h)			
	Normetanephrine	284.4	48.4	<76.9
	Epinephrine	452.6	1.3	<11.0
	MN (nmol/L)	48.52	0.37	<0.5
	NMN (nmol/L)	25.4	0.04	<0.9
	ACTH (pg/mL)	320	78.1	7.2–63.3
	Cortisol (μg/dL)	64.4	17.2	4.0–22.3
	24-h UFC (μg/24 h)	1883.3	73.8	12.3–103.5
B	24-h CAs in urine(μg/24 h)			
	Normetanephrine	39.3	3.4	≤106.8
	Epinephrine	11.6	1.3	≤15.3
	MN (nmol/L)	0.24	0.1	≤0.32
	NMN (nmol/L)	0.56	0.07	≤1.05
	ACTH (pg/mL)	273	<3	7.2–63.3
	Cortisol (μg/dL)	219.7	23	4.0–22.3
	24-h UFC (μg/24 h)	13,164	875.2	13.2–77.2
C	24-h CAs in urine(μg/24 h)			
	Normetanephrine	1185.5	69.6	≤106.8
	Epinephrine	320.9	0.9	≤15.3
	MN (nmol/L)	3.92	0.12	≤0.32
	NMN (nmol/L)	5.23	0.04	≤1.05
	ACTH (pg/mL)	1180	29.2	7.2–63.3
	Cortisol (μg/dL)	101.3	12.2	4.0–22.3
	24-h UFC (μg/24 h)	21,013.2	/	13.2–77.2

Abbreviations: MN: metanephrine, NMN: normetanephrine, ACTH: adrenocorticotropic hormone, 24-h UFC: 24-h urinary free cortisol. ACTH and serum cortisol levels were measured from morning blood samples collected at approximately 08:00 AM.

## Data Availability

The datasets presented in this article are not readily available because of privacy restrictions on Peking Union Medical Hospital’s database. Requests to access the datasets should be directed to beijingzhangyushi@126.com.

## Supplementary Materials

The following supporting information can be downloaded at: https://www.mdpi.com/article/10.3390/ijms27083625/s1.

## References

[1] Ariton M., Juan C.S., AvRuskin T.W. (2000). Pheochromocytoma: Clinical observations from a Brooklyn tertiary hospital. Endocr. Pract..

[2] Falhammar H., Kjellman M., Calissendorff J. (2018). Initial clinical presentation and spectrum of pheochromocytoma: A study of 94 cases from a single center. Endocr. Connect..

[3] Reisch N., Peczkowska M., Januszewicz A., Neumann H.P.H. (2006). Pheochromocytoma: Presentation, diagnosis and treatment. J. Hypertens..

[4] Lima J.V., Kater C.E. (2023). The Pheochromocytoma/Paraganglioma syndrome: An overview on mechanisms, diagnosis and management. Int. Braz. J. Urol..

[5] Negro A., Verzicco I., Tedeschi S., Campanini N., Zanelli M., Negri E., Farnetti E., Nicoli D., Palladini B., Santi R. (2021). Case Report: Irreversible Watery Diarrhea, Severe Metabolic Acidosis, Hypokalemia and Achloridria Syndrome Related to Vasoactive Intestinal Peptide Secreting Malignant Pheochromocytoma. Front. Endocrinol..

[6] Yee S.K., Meyer J.H., Wong L.L. (2022). Vasoactive Intestinal Peptide-Secreting Pheochromocytoma: A Case Report and Review of Literature. AACE Clin. Case Rep..

[7] Elliott P.F., Berhane T., Ragnarsson O., Falhammar H. (2021). Ectopic ACTH- and/or CRH-Producing Pheochromocytomas. J. Clin. Endocrinol. Metab..

[8] Huang J., Lin C., Fang L., Gu L., Cai X., Zeng H., Chen K., Mu Y., Liu J. (2025). Ectopic ACTH Syndrome Caused by Pheochromocytoma: A Case Report and Systematic Review. Endocr. Metab. Immune Disord. Drug Targets.

[9] Nakahira H., Miyauchi S., Watanabe K., Akesaka K., Ono K., Ebisui O., Miyake T., Furukawa S., Hiasa Y., Matsuura B. (2025). Pheochromocytoma associated with ectopic ACTH-producing tumor and hyper-interleukin-6emia: A case report with review of literature. Endocr. J..

[10] Ragnarsson O., Juhlin C.C., Torpy D.J., Falhammar H. (2024). A clinical perspective on ectopic Cushing’s syndrome. Trends Endocrinol. Metab..

[11] Ilias I., Torpy D.J., Pacak K., Mullen N., Wesley R.A., Nieman L.K. (2005). Cushing’s syndrome due to ectopic corticotropin secretion: Twenty years’ experience at the National Institutes of Health. J. Clin. Endocrinol. Metab..

[12] Isidori A.M., Kaltsas G.A., Pozza C., Frajese V., Newell-Price J., Reznek R.H., Jenkins P.J., Monson J.P., Grossman A.B., Besser G.M. (2006). The ectopic adrenocorticotropin syndrome: Clinical features, diagnosis, management, and long-term follow-up. J. Clin. Endocrinol. Metab..

[13] Kishlyansky D., Leung A.A., Pasieka J.L., Mahajan A., Kline G.A. (2024). Cushing syndrome from an ACTH-producing pheochromocytoma or paraganglioma: Structured review of 94 cases. Endocr. Relat. Cancer.

[14] Qin S., Xu Y., Yu S., Han W., Fan S., Ai W., Zhang K., Wang Y., Zhou X., Shen Q. (2024). Molecular classification and tumor microenvironment characteristics in pheochromocytomas. Elife.

[15] Wang M., Zheng G., Hu X., Tian F., Li T., Zhang Z., Gong K., Chen S., Yuan L., Qi Y. (2025). Single-Cell Atlas Reveals Tumorigenic Profiles and Immune Dynamics of Adrenal Incidentalomas. Adv. Sci..

[16] Berends A.M.A., Wardenaar R., van den Bos H., Tijhuis A.E., Links T.P., Feelders R.A., Hofland L.J., Kruijff S., Pacak K., Spierings D.C.J. (2025). Single-cell chromosome and bulk transcriptome analysis as a diagnostic tool to differentiate between localized and metastatic pheochromocytoma and sympathetic paraganglioma. Oncogene.

[17] Zhang X., Lian P., Su M., Ji Z., Deng J., Zheng G., Wang W., Ren X., Jiang T., Zhang P. (2021). Single-cell transcriptome analysis identifies a unique tumor cell type producing multiple hormones in ectopic ACTH and CRH secreting pheochromocytoma. Elife.

[18] Rindi G., Mete O., Uccella S., Basturk O., La Rosa S., Brosens L.A.A., Ezzat S., de Herder W.W., Klimstra D.S., Papotti M. (2022). Overview of the 2022 WHO Classification of Neuroendocrine Neoplasms. Endocr. Pathol..

[19] Mete O., Erickson L.A., Juhlin C.C., de Krijger R.R., Sasano H., Volante M., Papotti M.G. (2022). Overview of the 2022 WHO Classification of Adrenal Cortical Tumors. Endocr. Pathol..

[20] Mete O., Asa S.L., Gill A.J., Kimura N., de Krijger R.R., Tischler A. (2022). Overview of the 2022 WHO Classification of Paragangliomas and Pheochromocytomas. Endocr. Pathol..

[21] Vander Heiden M.G., Cantley L.C., Thompson C.B. (2009). Understanding the Warburg effect: The metabolic requirements of cell proliferation. Science.

[22] Pavlova N.N., Thompson C.B. (2016). The Emerging Hallmarks of Cancer Metabolism. Cell Metab..

[23] Jochmanova I., Pacak K. (2016). Pheochromocytoma: The First Metabolic Endocrine Cancer. Clin. Cancer Res..

[24] Rao J.U., Engelke U.F.H., Rodenburg R.J.T., Wevers R.A., Pacak K., Eisenhofer G., Qin N., Kusters B., Goudswaard A.G., Lenders J.W.M. (2013). Genotype-specific abnormalities in mitochondrial function associate with distinct profiles of energy metabolism and catecholamine content in pheochromocytoma and paraganglioma. Clin. Cancer Res..

[25] MacDonald P.E., Joseph J.W., Rorsman P. (2005). Glucose-sensing mechanisms in pancreatic beta-cells. Philos. Trans. R. Soc. B Biol. Sci..

[26] Calsina B., Piñeiro-Yáñez E., Martínez-Montes ÁM., Caleiras E., Fernández-Sanromán Á, Monteagudo M., Torres-Pérez R., Fustero-Torre C., Pulgarín-Alfaro M., Gil E. (2023). Genomic and immune landscape Of metastatic pheochromocytoma and paraganglioma. Nat. Commun..

[27] Chang C.-H., Qiu J., O’Sullivan D., Buck M.D., Noguchi T., Curtis J.D., Chen Q., Gindin M., Gubin M.M., van der Windt G.J.W. (2015). Metabolic Competition in the Tumor Microenvironment Is a Driver of Cancer Progression. Cell.

[28] Jin S., Plikus M.V., Nie Q. (2025). CellChat for systematic analysis of cell-cell communication from single-cell transcriptomics. Nat. Protoc..

[29] Fishbein L., Leshchiner I., Walter V., Danilova L., Robertson A.G., Johnson A.R., Lichtenberg T.M., Murray B.A., Ghayee H.K., Else T. (2017). Comprehensive Molecular Characterization of Pheochromocytoma and Paraganglioma. Cancer Cell.

[30] (2025). Transparency in the Reporting of Artificial INtelligence—The TITAN Guideline—Premier Science. https://premierscience.com/pjs-25-950/.

