# Supplementary material for: Single-Cell Transcriptomic Profiling of Ectopic ACTH-Secreting Pheochromocytoma Reveals the Chromaffin Cell Origin of Ectopic Hormone Production
Source: Int J Mol Sci. 2026 Apr 18;27(8):3625. doi: 10.3390/ijms27083625 (PMC13116716; doi:10.3390/ijms27083625)
Supplement: Supplementary file 1 [file ijms-27-03625-s001.zip › Supplementary Figures.pdf]

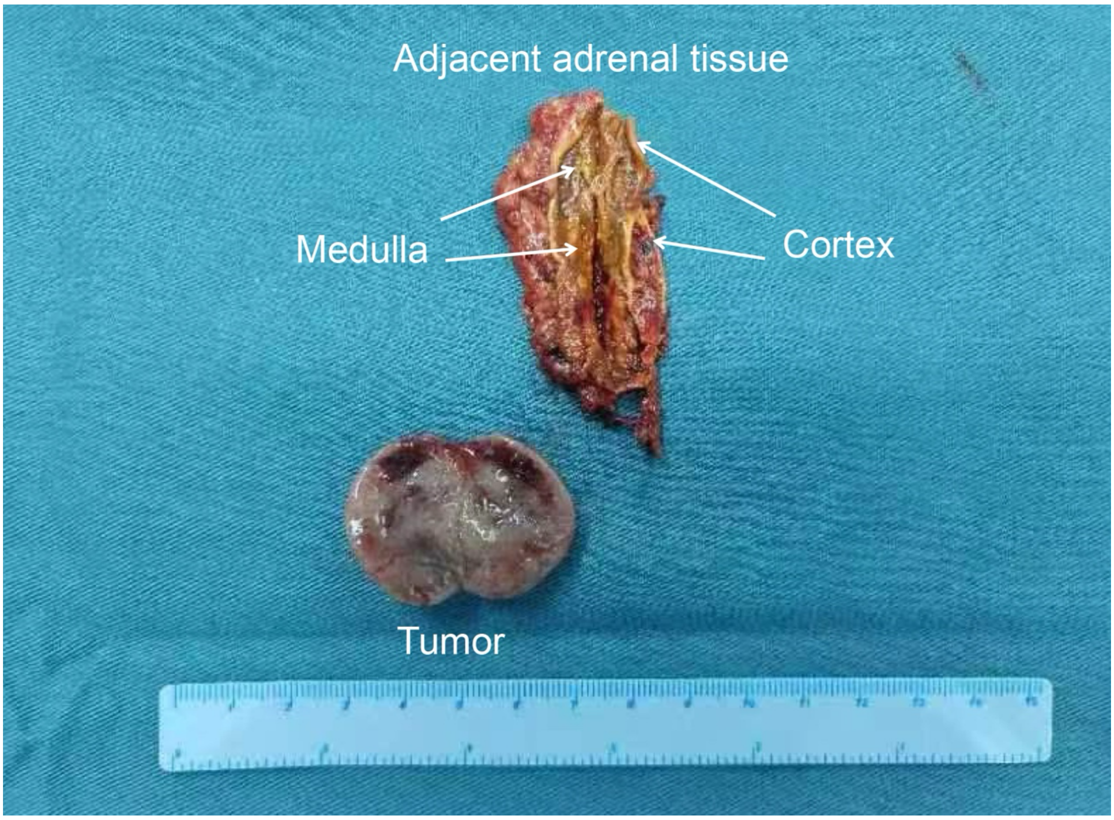

Supplementary Figure S1. Gross specimen of tumor and adjacent adrenal tissue

Representative gross specimen obtained during surgical resection demonstrating the pheochromocytoma tumor mass and adjacent adrenal tissue. The residual adrenal medulla (brown to reddish-brown central region) and the surrounding adrenal cortex (yellow to tan peripheral region) are indicated by arrows, consistent with their typical macroscopic anatomical appearance.

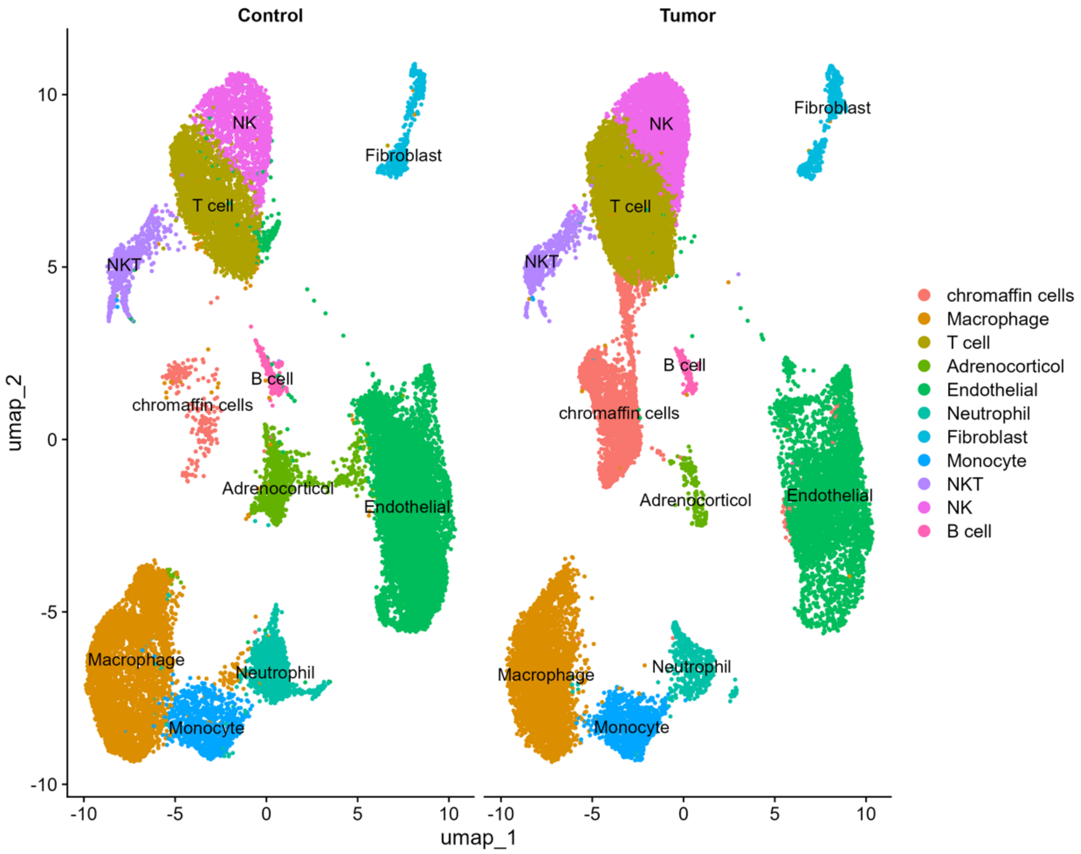

Supplementary Figure S2. Distribution of tumor and adjacent adrenal cells in the integrated UMAP space.

Cells are colored according to sample origin (tumor vs adjacent adrenal tissue). Chromaffin cell populations can be observed in both tumor-derived and adjacent adrenal samples.

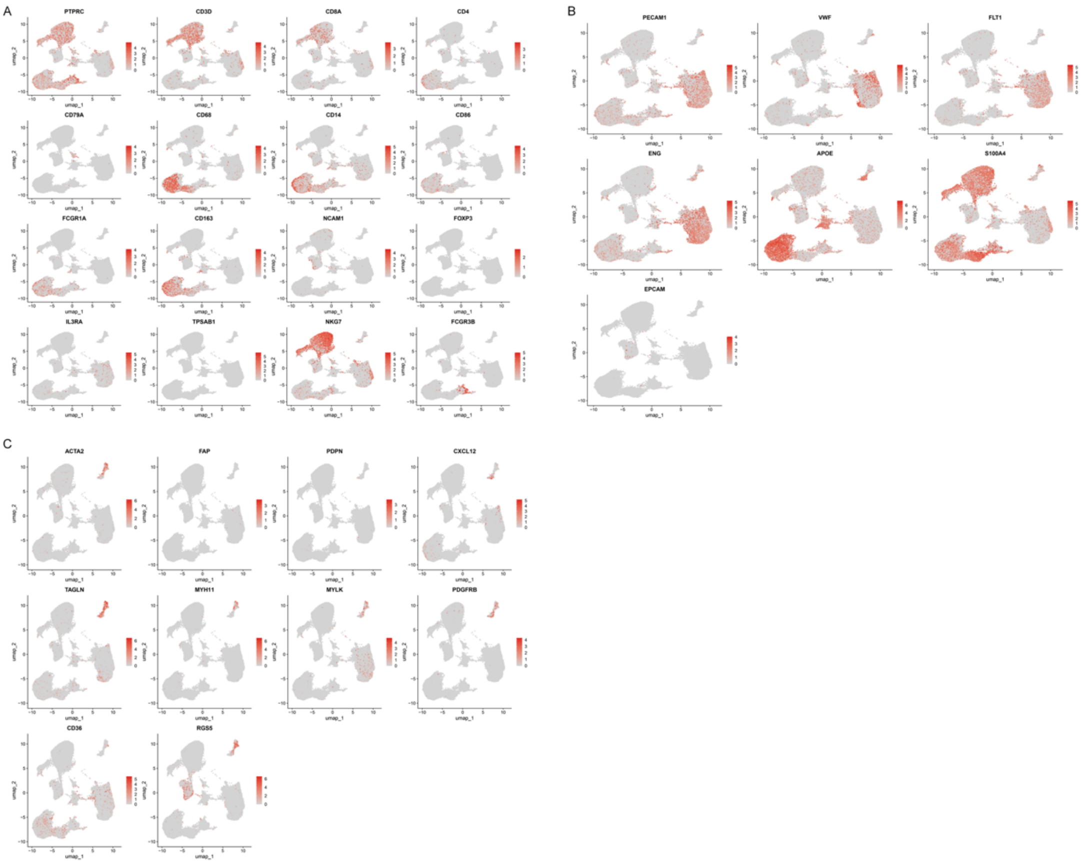

Supplementary Figure S3 Annotation of Additional Cell Clusters

FeaturePlots visualizing the expression of key marker genes used to identify and annotate the non-steroidogenic and non-chromaffin cell populations within the eas-PCC. The markers confirmed the presence of immune (T, B, NK, monocyte) and stromal (endothelial, fibroblast) populations within the TME.

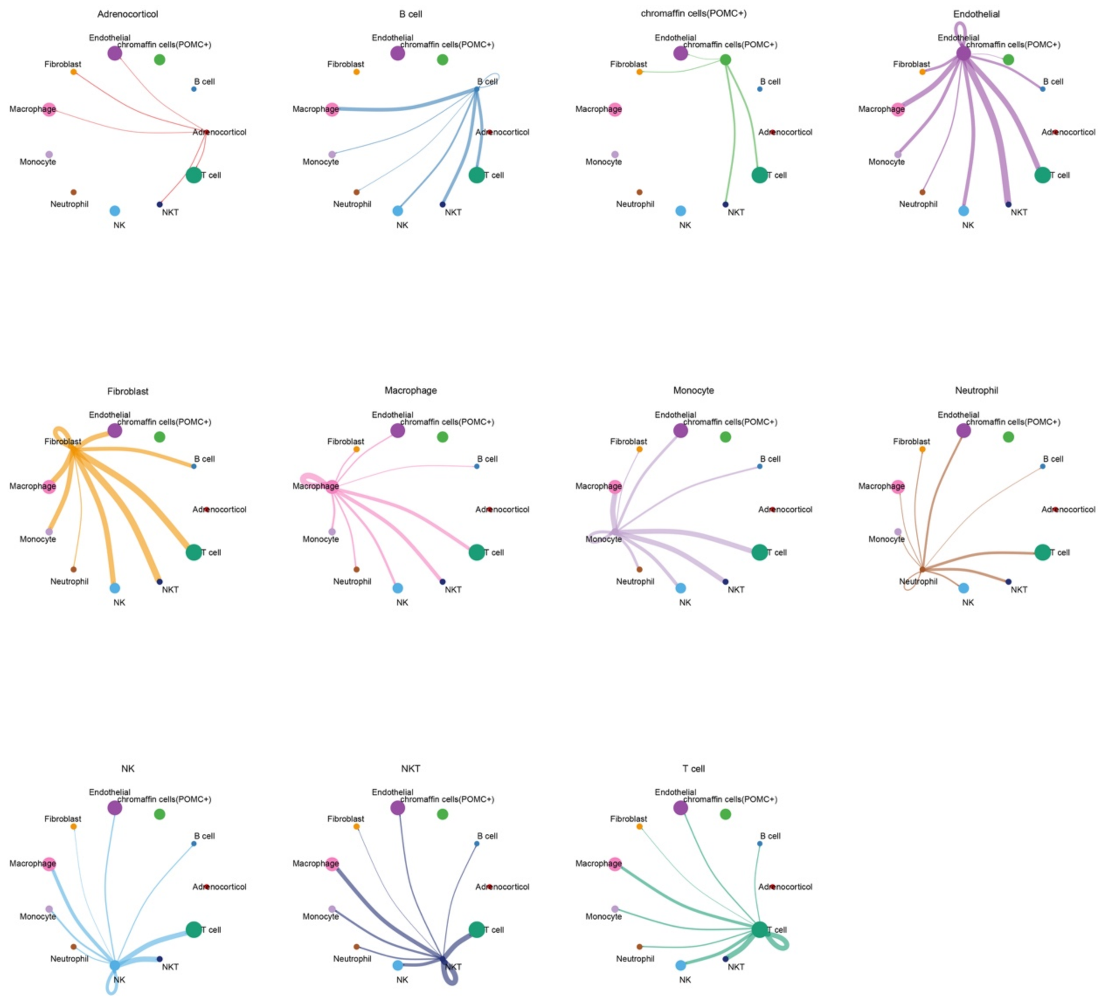

Supplementary Figure S4. Cluster-Specific Overview of Cell-Cell Communication.
Circle plots visualizing the outgoing and incoming interaction strengths for each of the 11 annotated cell clusters. For each cluster, the communication patterns were displayed separately. The line width connecting the clusters represented the number of interactions.
