# Supplementary material for: Single-Cell Transcriptomic Profiling of Ectopic ACTH-Secreting Pheochromocytoma Reveals the Chromaffin Cell Origin of Ectopic Hormone Production
Source: Int J Mol Sci. 2026 Apr 18;27(8):3625. doi: 10.3390/ijms27083625 (PMC13116716; doi:10.3390/ijms27083625)
Supplement: Supplementary file 1 [file ijms-27-03625-s001.zip › Supplementary Table S1.pdf]

Supplementary Table S1 The signature of catecholamine and corticosteroid

Gene symbol	Full Name
TH	Tyrosine Hydroxylase
DBH	Dopamine Beta-Hydroxylase
PNMT	Phenylethanolamine N-Methyltransferase
CHGA	Chromogranin A
CHGB	Chromogranin B
SCG2	Secretogranin II
SCG3	Secretogranin III
SCG5	Secretogranin V
SYN	Synaptophysin
SLC18A1	Solute Carrier Family 18 Member A1
SLC18A2	Solute Carrier Family 18 Member A2
SYT1	Synaptotagmin 1
CYP11B2	Cytochrome P450 Family 11 Subfamily B Member 2
CYP21A2	Cytochrome P450 Family 11 Subfamily A Member 2
CYP17A1	Cytochrome P450 Family 17 Subfamily A Member 1
STAR	Steroidogenic Acute Regulatory Protein
HSD3B2	Hydroxy-delta-5-steroid Dehydrogenase, 3-beta- and Steroid delta-isomerase 2
FDX1	Ferredoxin 1
MC2R	Melanocortin 2 Receptor
POR	Cytochrome P450 Oxidoreductase
SULT2A1	Sulfotransferase Family 2A Member 1
HSD17B12	Hydroxysteroid 17-Beta Dehydrogenase 12
CRH	Corticotropin-Releasing Hormone
AVP	Arginine Vasopressin
