# Supplementary material for: Single-Cell Transcriptomic Profiling of Ectopic ACTH-Secreting Pheochromocytoma Reveals the Chromaffin Cell Origin of Ectopic Hormone Production
Source: Int J Mol Sci. 2026 Apr 18;27(8):3625. doi: 10.3390/ijms27083625 (PMC13116716; doi:10.3390/ijms27083625)
Supplement: Supplementary file 1 [file ijms-27-03625-s001.zip › Supplementary Table S2.pdf]

Supplementary Table S2 The markers of cell annotation

Cell type	Markers
T cell	CD3D, CD4, CD8A
B cell	CD79A
CHGA+/ACTH+ cell	TH, DBH, CHGA,CHGB,POMC
Adrenocorticol	CYP17A1,CYP21A2,STAR
Endothelial	PECAM1,ENG,VWF
Macrophage	CD68,CD163,CD206
NK cell	NCAM1, NKG7, KLRF1
Monocyte	CD14、FCN1、LYZ、S100A8
Neutrophil	FCGR3B
NKT cell	KLRB1, CD3D, NKG7
Fibroblast	FAP, COL1AFAP, COL1A1, DCN, PDGFRA1
